# Microsatellite Instability, Epstein–Barr Virus, and Programmed Cell Death Ligand 1 as Predictive Markers for Immunotherapy in Gastric Cancer

**DOI:** 10.3390/cancers14010218

**Published:** 2022-01-03

**Authors:** Hung-Yuan Yu, Chung-Pin Li, Yi-Hsiang Huang, Shao-Jung Hsu, Yen-Po Wang, Yun-Cheng Hsieh, Wen-Liang Fang, Kuo-Hung Huang, Anna Fen-Yau Li, Rheun-Chuan Lee, Kang-Lung Lee, Yuan-Hung Wu, I-Chun Lai, Wan-Chin Yang, Yi-Ping Hung, Yu-Chao Wang, Shu-Hui Chen, Ming-Huang Chen, Yee Chao

**Affiliations:** 1 Division of Gastroenterology and Hepatology, Department of Medicine, Taipei Veterans General Hospital, Taipei 112201, Taiwan; hyyu6@vghtpe.gov.tw (H.-Y.Y.); cpli@vghtpe.gov.tw (C.-P.L.); yhhuang@vghtpe.gov.tw (Y.-H.H.); sjhsu@vghtpe.gov.tw (S.-J.H.); ypwang@vghtpe.gov.tw (Y.-P.W.); ychsieh7@vghtpe.gov.tw (Y.-C.H.); 2 Hospitalist Ward, Department of Medicine, Taipei Veterans General Hospital, Taipei 112201, Taiwan; 3 School of Medicine, College of Medicine, National Yang Ming Chiao Tung University, Taipei 112201, Taiwan; wlfang@vghtpe.gov.tw (W.-L.F.); khhuang@vghtpe.gov.tw (K.-H.H.); fyli@vghtpe.gov.tw (A.F.-Y.L.); rclee@vghtpe.gov.tw (R.-C.L.); kllee2@vghtpe.gov.tw (K.-L.L.); yhwu13@vghtpe.gov.tw (Y.-H.W.); iclai2@vghtpe.gov.tw (I.-C.L.); wcyang3@vghtpe.gov.tw (W.-C.Y.); yphong@vghtpe.gov.tw (Y.-P.H.); 4 Division of Clinical Skills Training, Department of Medical Education, Taipei Veterans General Hospital, Taipei 112201, Taiwan; 5 Division of General Surgery, Department of Surgery, Taipei Veterans General Hospital, Taipei 112201, Taiwan; 6 Department of Pathology, Taipei Veterans General Hospital, Taipei 112201, Taiwan; 7 Department of Radiology, Taipei Veterans General Hospital, Taipei 112201, Taiwan; 8 Department of Oncology, Taipei Veterans General Hospital, Taipei 112201, Taiwan; 9 Institute of Biomedical Informatics, National Yang Ming Chiao Tung University, Taipei 112304, Taiwan; yuchao@ym.edu.tw; 10 Department of Nursing, Taipei Veterans General Hospital, Taipei 112201, Taiwan; sh_chen@vghtpe.gov.tw

**Keywords:** microsatellite instability, Epstein–Barr virus, programmed cell death ligand 1, gastric cancer, immunotherapy

## Abstract

**Simple Summary:**

Immunotherapy is approved in selected cases of gastric cancer, and durable responses have been observed in exceptional responders. Several potential predictive biomarkers have been identified in gastric cancer, such as microsatellite instability-high (MSI-H), Epstein–Barr virus (EBV), and programmed death ligand 1 (PD-L1). We explored the real-world evidence of these biomarkers and their outcomes. When only combined positive score (CPS) ≥ 1 was used as the biomarker, the overall response rate (ORR) and progression-free survival (PFS) were not statistically significant. CPS ≥ 1 was commonly combined with MSI-H (75%) and Epstein–Barr encoding region (EBER) (80%). MSI-H and CPS ≥ 5 were prognostic biomarkers associated with better ORR and PFS. In patients with EBER, better ORR and PFS were observed only in patients with CPS ≥ 1. These results could transform clinical practice and can be used to formulate more precise treatment suggestions for patients with gastric cancer.

**Abstract:**

Immunotherapy benefits selected cases of gastric cancer (GC), but the correlation between biomarkers and prognosis is still unclear. Fifty-two patients with GC who underwent immunotherapy were enrolled from June 2016 to December 2020. Their clinical features and biomarkers—microsatellite instability-high (MSI-H), programmed cell death ligand 1 (PD-L1) combined positive score (CPS), and Epstein–Barr encoding region (EBER)—were analyzed. Eight patients had MSI-H, five patients had EBER, 29 patients had CPS ≥ 1, and 20 patients had no biomarker. The overall response rates (ORRs) of the MSI-H, EBER, PD-L1 CPS ≥ 1, and all-negative group were 75%, 60%, 44.8%, and 15%, respectively. Compared with that of the all-negative group, progression-free survival (PFS) was better in the MSI-H (*p* = 0.018), CPS ≥ 5 (*p* = 0.012), and CPS ≥ 10 (*p* = 0.006) groups, but not in the EBER (*p* = 0.2) and CPS ≥ 1 groups (*p* = 0.35). Ten patients had combined biomarkers, CPS ≥ 1 with either MSI-H or EBER. The ORRs were 66.7% for CPS ≥ 1 and MSI-H and 75% for CPS ≥ 1 and EBER. PFS was better in patients with combined biomarkers (*p* = 0.01). MSI-H, EBER, and CPS are useful biomarkers for predicting the efficacy of immunotherapy.

## 1. Introduction

Gastric adenocarcinoma is a life-threatening cancer with high incidence and mortality rates. Despite its decreasing annual incidence, gastric adenocarcinoma is still the fifth most common cancer and fourth leading cause of cancer death [1]. Systemic therapy or chemoradiation remains the standard first-line treatment in selected cases of locally advanced or metastatic gastric cancers. However, despite current treatments, the 5-year survival rate is still low, and a large unmet need still exists for the treatment of advanced or metastatic gastric cancer.

The etiology of gastric cancer (GC) is heterogeneous, and The Cancer Genome Atlas (TCGA) project has classified GCs into four subtypes according to their molecular presentations [2]: tumors positive for Epstein–Barr virus (EBV), microsatellite-unstable tumors, genomically stable tumors, and tumors with chromosomal instability. These molecular alterations could affect different pathways of cancer development, resulting in different outcomes in patients with GC undergoing immunotherapy. Several studies have investigated the relationship of biomarkers, such as Epstein–Barr encoding region (EBER) [3,4], microsatellite instability-high (MSI-H) [5,6], and programmed cell death ligand 1 (PD-L1), as well as their associated outcomes in patients intended for immunotherapy [7,8].

EBV is one of the human herpes viruses implicated in the etiology of several malignancies, including GCs [9,10]. EBV-associated gastric carcinoma (EBVaGC) is a distinct subtype that is defined by monoclonal proliferation of carcinoma cells with latent EBV infection. EBER in situ hybridization is a method for detecting EBV in tissue sections. Clinical characteristics of EBVaGC include male predominance, younger age, proximal location in the stomach, lymphoepithelioma-like histology [11], and favorable prognosis [12]. EBVaGC develops owing to both genetic and epigenetic changes caused by EBV infection, which may result in favorable outcomes following immunotherapy [3,13].

Microsatellite instability-high (MSI-H) is a subtype of GC characterized by DNA mismatch repair deficiency (dMMR) or microsatellite instability (MSI). MMR deficiency resulting from mutational inactivation or epigenetic silencing of DNA mismatch repair genes (e.g., MSH2, MSH3, MSH6, MLH1, and PMS2) causes MSI, which is characterized by alteration in the length of short, repeated DNA sequences (microsatellites), possibly resulting in hypermutation in cancer cells and the expression of abundant peptides that function as neoantigens [6,10]. MSI-H GCs are usually associated with female sex, older age, distal location, no lymph node involvement, intestinal type, lower local invasion capacity, earlier stage, and better survival [14]. A previous study reported that treatment with immune check-point inhibitors (ICIs) in MSI-H GC is associated with better prognosis [5]. This phenomenon may result from hypermutated phenotypes expressing abundant peptides that could trigger a patient’s immune system when inhibiting the programmed cell death (PD-1)/PD-L1 pathway [6].

ICIs are the most common immunotherapy for cancer treatment. Common ICIs include anti-PD-1 antibody, anti-PD-L1 antibody, and anti-cytotoxic T-lymphocyte antigen 4 (CTLA-4) antibody. These agents have been widely used for treating several cancers, including melanoma, bladder cancer, renal cell cancer, non-small-cell lung cancer, and gastrointestinal cancers. Cancer cells escape the immune system, creating an immunosuppressive environment by overexpressing PD-L1 on their cell surfaces or inducing PD-L1/CTLA-4 expression on immune cells. ICIs could block this pathway and enhance the immune response. However, because ICIs do not always produce better outcomes, biomarkers are required to identify patients with better responses to ICIs. Several biomarkers were identified for prediction, such as PD-L1, MSI-H and EBER. Other than these typical biomarkers, other non-typical biomarkers were also investigated, such as the B7 family [15]. PD-L1 expression was the first potential biomarker identified for predicting the response to ICIs, and tumor proportion score (TPS) was introduced as a prognostic factor in patients with non-small-cell lung cancer receiving pembrolizumab [16]. Further data have indicated that PD-L1 staining on tumor-associated immune cells is as important as staining on the tumor. Hence, the combined positive score (CPS) was developed to predict the outcomes of ICIs in patients with GC [17]. MSI-H was also a widely used biomarker for immunotherapy. Several studies and post-hoc analysis showed great correlation between MSI-H and better clinical outcomes. EBER was also a potential biomarker for outcome prediction. Despite previous studies demonstrating better outcomes in EBV-positive patients, recent studies showed no statistical significance, with some studies reporting conflicting results. Therefore, the roles of these biomarkers should be further evaluated.

Although several biomarkers have been identified for predicting outcomes in patients with GC receiving ICIs, only a few studies have discussed the relationship between these biomarkers. Therefore, in this study, we explored the real-world experience of immunotherapy in patients with GC, the relationship between different biomarkers, and clinical outcomes.

## 2. Materials and Methods

### 2.1. Study Design and Participants

This retrospective study enrolled all patients with gastric adenocarcinoma who received ICIs at Taipei Veterans General Hospital between June 2016 and October 2020, including patients with ICI monotherapy, combined immunotherapy and chemotherapy, and combined immunotherapy. This study was approved by the Institutional Review Board of Taipei Veterans General Hospital (2019-10-005AC) and followed the tenets of the Helsinki Declaration. Basic characteristics, including age, sex, ECOG, liver function, renal function, initial staging, pathological finding, treatment courses, and previous treatment, were recorded.

### 2.2. Investigation of Potential Biomarkers for Immunotherapy

Informed consent forms were signed, and the previous biopsy or surgical resection samples were sent for further immunohistochemical (IHC) stain, including MLH1, MSH2, MSH6, and PMS2 for mismatch repair protein; Epstein–Barr virus-encoded regions (EBER) in situ hybridization (ISH) was performed using the EBV Probe/Antibody ISH Kit (Leica Biosystems Newcastle Ltd., Newcastle-upon-Tyne, UK) in association with Ultra Vision Large Volume Detection System Anti-Polyvalent, HRP (Thermo Fisher Scientific, Fremont, CA, USA), which served as a gold standard to define EBV-associated GC [10]. PD-L1 was evaluated through the pharmDx immunohistochemistry assay (PD-L1 IHC 22C3) combined positive score (CPS).

### 2.3. Clinical Response, Durations of Response, and Survival Analysis

Tumor size was measured using computed tomography (CT) or magnetic resonance imaging (MRI), with a follow-up interval of 3 months; this interval may be adjusted if clinically indicated. The clinical response was evaluated on the basis of response evaluation criteria in solid tumors (RECIST), version 1.1. Duration of response was defined as the duration from initial response to disease progression. Survival analysis was performed using the Kaplan–Meier method.

### 2.4. Tumor Mutation Burden (TMB) and Tumor Infiltrating Lymphocytes (TILs) Analysis Using TCGA Database

For gastric cancer patients with RNA-seq gene expression in TCGA database, the relationship of EBV and PD-L1 was investigated. The EBV status of each patient was determined based on the EBV molecular subtype [2]. Moreover, the samples with the top 25% of mRNA expression of PD-L1 were defined as PD-L1 high according to a previous study [18]. Consequently, the predictive biomarkers such as TMB and TILs, which were estimated using MCP-counter [19], were compared between EBV-positive/PD-L1 high samples and EBV-positive/PD-L1 low samples using the Mann–Whitney U test. TMB was defined as the total number of mutations in a sample that can be obtained from TCGA multicenter mutation calling in multiple cancers (MC3) project [20].

### 2.5. Statistical Analysis

Baseline characteristics were summarized as median (range) of continuous variables and absolute numbers (proportions) of categorical variables. The relationship between clinical response and biomarkers were analyzed using χ2 test and Fisher’s exact test. Survival analyses were performed using the Kaplan–Meier method, and significance was analyzed using the log-rank test. Statistical significance was defined as *p* < 0.05.

## 3. Results

### 3.1. Patient Characteristics

A total of 52 patients were enrolled in this study, and their baseline characteristics are summarized in Table 1. The mean age was 65.5 years, and 23 patients were male (44.2%). Most patients were Eastern Cooperative Oncology Group (ECOG) performance status 0–1 (92.3%).

Eight patients had MSI-H, five patients had EBER, 29 patients had PD-L1 CPS ≥ 1, and 20 patients had no biomarker. Regarding different cut-off values, nine patients had CPS ≥ 5, and six patients had CPS ≥ 10. Combined biomarkers were observed in our study. 

Immunotherapy was used as first-line therapy in 10 patients (19.2%), second-line therapy in 10 patients (19.2%), and third- or later-line therapy in 32 patients (61.5%). Thirty-eight patients (73.1%) were treated with nivolumab; 12 patients (23.1%) with pembrolizumab; and two patients (3.8%) with atezolizumab. Among these patients, 45 patients received immunotherapy monotherapy, two patients received combined therapy comprising two types of immunotherapies, and five patients were treated with immunotherapy and chemotherapy.

### 3.2. Overall Response Rates (ORRs) and Progression-Free Survival (PFS) in Patients with Different Biomarkers

**Figure 1:**
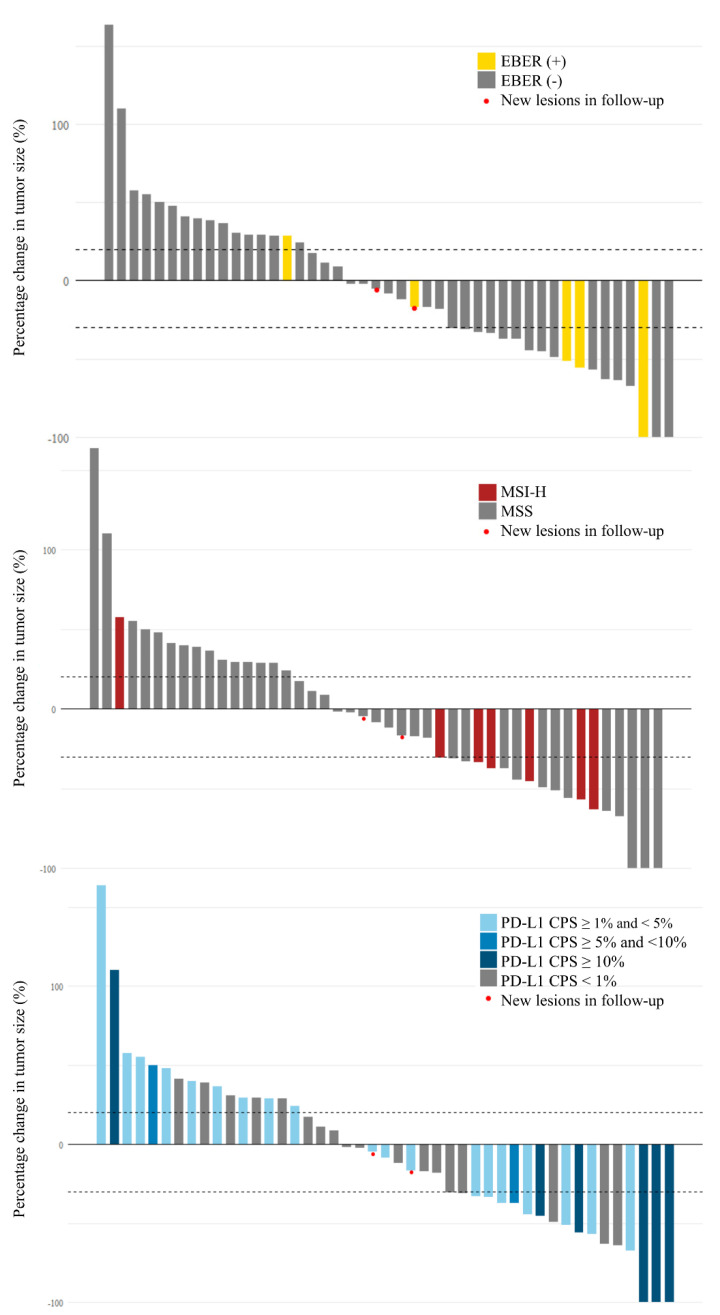
Waterfall plot for percentage change in tumor size with different biomarkers. EBER: Epstein–Barr virus-encoded small RNAs; MSI-H: high microsatellite instability; PD-L1: programmed death ligand 1; CPS: combined positive score; PD: progressive disease.

The ORRs and percentage change in the tumor size of patients with MSI-H, PD-L1 CPS ≥ 1, and EBER are summarized in Table 2 and Figure 1, respectively. The ORRs of the MSI-H, EBER, PD-L1 CPS ≥ 1, and all-negative groups were 75%, 60%, 44.8%, and 15%, respectively. The ORRs of the MSI-H, and PD-L1 CPS ≥ 1 group were significantly higher than those of the all-negative group (*p* = 0.035, 0.005, respectively), but the finding in the EBER group did not reach statistical significance (*p* = 0.07).

**Figure 2:**
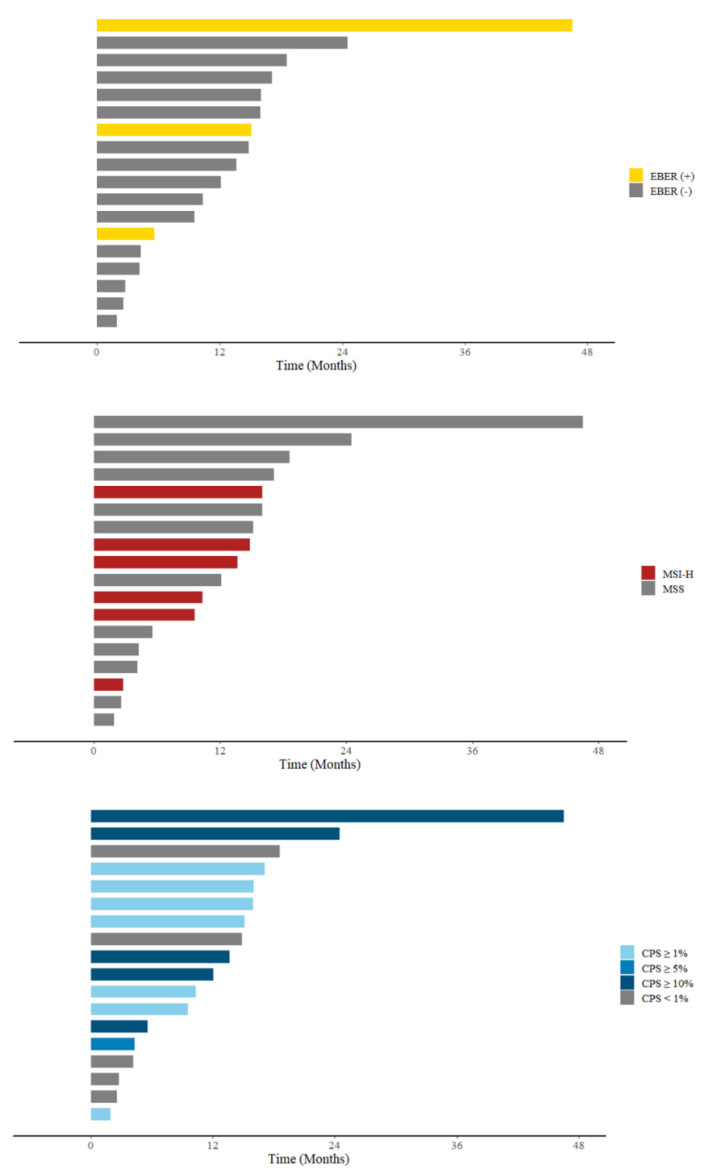
Timeline plot for durations of response, including patients with complete response and partial response. EBER: Epstein–Barr virus-encoded small RNAs; MSI-H: high microsatellite instability; PD-L1: programmed death ligand 1; CPS: combined positive score; PD: progressive disease.

**Figure 3:**
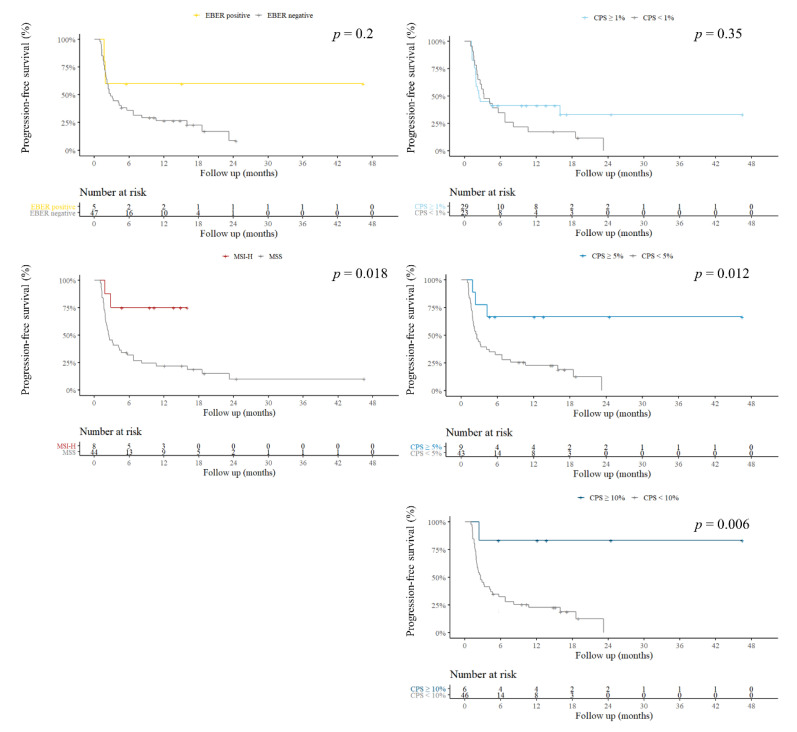
Survival analysis in patients with and without each biomarker. CPS: combined positive score; EBER: Epstein–Barr virus (EBV)-encoded small RNAs; MSI-H: high microsatellite instability; PD-L1: programmed death ligand 1.

The durations of the response and PFS in patients with different biomarkers are summarized in Figure 2 and Figure 3, respectively. PFS was 3.2 months in the all-negative group. Compared with the all-negative group, PFS was significantly better in the MSI-H group (not reached vs. 3.2 months, *p* = 0.018). PFS in the EBER group (not reached vs. 3.2 months, *p* = 0.2) and CPS ≥ 1 group (2.4 months vs. 3.2 months, *p* = 0.35) were not better than that of the all-negative group.

### 3.3. ORRs and PFS in Different Cut-Off Levels of CPS (CPS ≥ 1, ≥ 5, and ≥ 10)

The ORR and percentage change in the tumor size of patients with different cut-off levels of PD-L1 are summarized in Table 3 and Figure 1, respectively. The ORRs of patients with CPS ≥ 1, ≥ 5, and ≥ 10 were 44.8%, 66.7%, and 83.3%, respectively. Higher percentage of PD-L1 CPS expression indicated more effective immunotherapy.

The durations of the response and PFS in patients with different biomarkers are summarized in Figure 2 and Figure 3. PFS was also significantly better in the CPS ≥ 5% (not reached vs. 3.2 months, *p* = 0.012) and CPS ≥ 10% (not reached vs. 3.2 months, *p* = 0.006) groups. No statistical significance was observed in the CPS ≥ 1 group (2.4 months vs. 3.2 months, *p* = 0.35).

### 3.4. Combined Biomarkers: Incidence, ORR, and PFS

**Figure 4:**
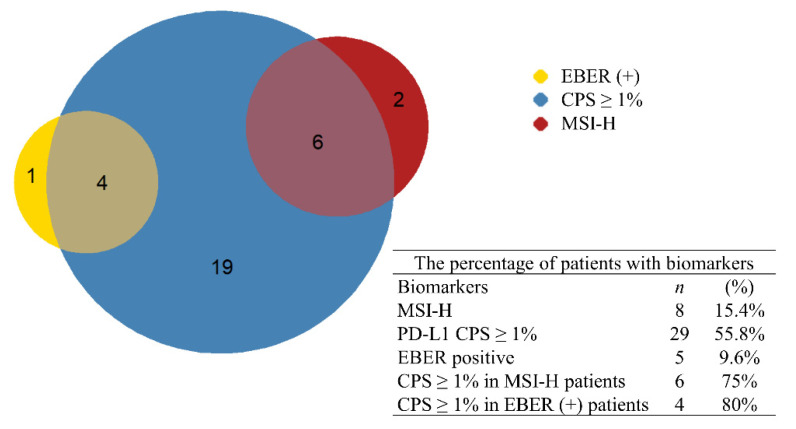
Incidence of biomarkers in gastric cancer patients. EBER: Epstein–Barr virus-encoded small RNAs; MSI-H: high microsatellite instability; PD-L1: programmed death ligand 1; CPS: combined positive score.

**Figure 5:**
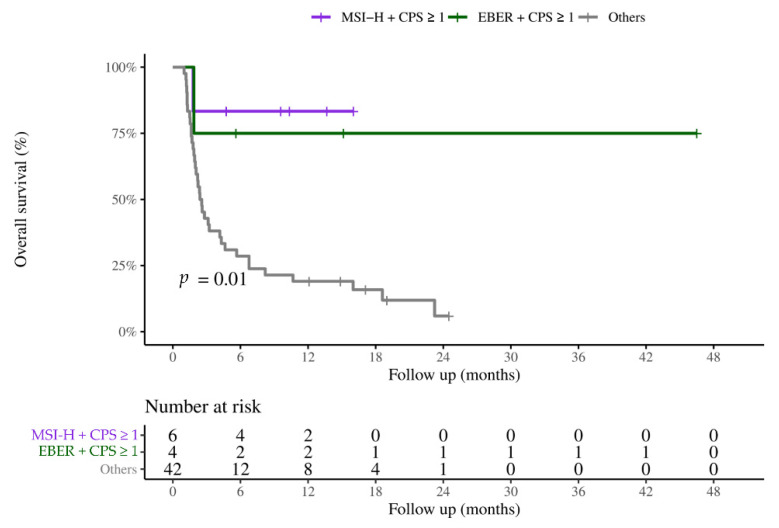
Survival curves of patients with combined biomarkers. EBER: Epstein–Barr virus-encoded small RNAs; MSI-H: high microsatellite instability; PD-L1: programmed death ligand 1; CPS: combined positive score.

Figure 4 illustrates the correlation among all biomarkers. Among all patients with MSI-H, six patients (75%) also had CPS ≥ 1. In addition, four patients (80%) had both EBER and CPS ≥ 1. No patient had both MSI-H and EBER, indicating that MSI-H and EBER could be mutually exclusive. In patients with combined biomarkers, the ORRs were 66.7% in patients with MSI-H and CPS ≥ 1 and 75% in patients with EBER and CPS ≥ 1. Patients with combined biomarkers also had more durable responses and better PFS (median PFS was not reached during follow-up) compared with other patients (Figure 5).

## 4. Discussion

In our study, the incidence of MSI-H (15.4%), EBV-positive (9.6%), and PD-L1 CPS ≥1 (55.8%) is similar to those of other studies [2,7,11]. The ORRs of patients with MSI-H, EBER, or PD-L1 CPS ≥ 1 tumors were better than those of the all-negative patients, and patients with higher PD-L1 CPS had better ORRs. Increased PFS was also observed in patients with either one or two biomarkers compared with the all-negative controls. These results are consistent with those of clinical trials. KEYNOTE-059 [7] reported promising efficacy and measurable safety for pembrolizumab in patients with PD-L1 CPS ≥ 1 (PD-L1 IHC 22C3) who had advanced gastric or gastro-esophageal junction cancer that progressed after second- or later-line treatment. KEYNOTE-062 [8] demonstrated that compared with chemotherapy, pembrolizumab monotherapy as first-line therapy produced a non-inferior response in patients with GC having PD-L1 CPS ≥ 1. Pembrolizumab also prolonged overall survival (OS) in patients with PD-L1 CPS ≥ 10 tumors. However, cross over was observed in the survival curves of OS. In the subgroup analysis, patients with MSI-H had better OS in KEYNOTE-062 [5]. Therefore, MSI-H and PD-L1 CPS could be valuable biomarkers for predicting the prognosis of pembrolizumab, but the cut-off value should be established. CheckMate-649 [21] demonstrated better prognosis for nivolumab combined with chemotherapy than for chemotherapy alone as the first-line therapy in patients with PD-L1 CPS ≥ 5 (Dako PD-L1 immunohistochemistry 28–8 pharmDx assay). These results were supported by ATTRACTION-04 [22], which revealed improvements in PFS with the combination of nivolumab and chemotherapy, although OS was not significantly altered. ATTRACTION-04 was designed for all-comers without regard to any specific biomarker, which may have resulted in the insignificant benefit of OS. ATTRACTION-02 [23] also demonstrated better OS for nivolumab monotherapy than for placebo in patients with progressive GC after two lines of therapy, independent from PD-L1 expression status. According to these trials, PD-L1 seems to be a suitable biomarker, with the strongest predictive value at CPS ≥ 10. Similar results were observed in the studies for nivolumab and pembrolizumab.

MSI-H is significantly associated with a long-term response and better prognosis in several types of malignancies, including GC [24]. In post hoc analyses of KEYNOTE-012, KEYNOTE-059, and KEYNOTE-158, the ORR of MSI-H patients was better than that of microsatellite-stable (MSS) patients (57.1%, 57.1%, and 46%, respectively). One meta-analysis of the predictive role of MSI-H in patients undergoing ICIs revealed a hazard ratio of 0.34 for the OS benefit (vs. 0.82 for MSS GC) for anti-PD-1 regimens compared with chemotherapy alone [5]. These studies all revealed promising ORR and survival in patients with MSI-H undergoing ICIs.

EBV is a herpesvirus that has been identified in the tumor cells of a heterogeneous group of malignancies [9]. EBV alters the human immune response by specific gene expression, miRNA, and DNA methylation [4]. EBV-positive GC is a unique subgroup with distinct oncogenesis, molecular profile, and clinical pathology. Previous studies have revealed that patients with EBV-positive GC had superior outcomes than EBV-negative patients [3,25]. Several theories have been proposed to explain this finding. First, studies have demonstrated that immune cell signaling is activated in EBV-positive GC [2]. Second, PD-L1 overexpression was observed in EBV-positive GC according to The Cancer Genome Atlas (TCGA) and other studies [2,26].

In this study, PFS was not statistically different between EBER-positive patients and PD-L1 CPS ≥ 1 patients. In these two groups, survival curves did not separate from that of the all-negative patients in the first 3 months. However, better PFS was noted after 3 months, suggesting that patients who did not respond to ICIs could not be identified using the biomarkers. This phenomenon was reported in previous clinical trials, such as KEYNOTE-062 [8]. Most patients with PD-L1 CPS ≥ 1 and stable disease or progression after treatment were sampled based on biopsy (12/16, 75%) rather than based on surgery and CPS < 5 in most patients (13/16, 81%). The small pathologic sample may be related to false positive and false negative PD-L1, which may lead to misclassification in non-small-cell lung cancers [27]. Furthermore, in CheckMate-649, superior OS and PFS were observed in patients with PD-L1 CPS ≥ 5 [21]. Therefore, PD-L1 CPS ≥ 5 is suggested as a biomarker of ICI therapy, and PD-L1 CPS ≥ 10 is associated with better prognosis.

Although recent studies have demonstrated better outcomes in EBV-positive patients, several studies have not reported statistical significance, with some studies reporting conflicting results [28,29]. A previous study reported poor prognosis in intestinal-type carcinoma with Lauren’s classification [30]. Fang et al. [10] reported the association of cancer types with PD-L1 expression and gene mutations. In EBV-positive GCs, higher PD-L1 expression was observed in intestinal or solid types, and more PI3K/AKT pathway mutations were identified in lymphoepithelioma-like GCs. This molecular difference may result in different response rates to ICIs in different cell types. Lee et al. [31] also noticed various treatment outcomes between EBV-positive patients with GC. They then divided all EBV-positive GC patients into two clusters for hierarchical cluster analysis according to the protein expression profile. Significantly better outcomes were observed in cluster 1, and the survival rate was worse in cluster 2 compared with cluster 1 and EBV-negative patients. However, EBV status was not associated with the patient survival rate, either in the intestinal type or in the diffuse type. Different downstream pathways or genetic events beyond EBV infection could result in different clinical outcomes. In summary, EBV-associated GC develops through various genetic and epigenetic alterations, and heterogeneous outcomes were also identified. However, currently, no biomarker can be used to definitively predict prognosis.

The relationship between biomarkers was also investigated. PD-L1 ≥ 1 was found in most EBV-positive patients (4/5) and MSI-H patients (6/8). No patient had both EBV-positive status and MSI-H, a finding that has also been reported in several studies [13,32,33]. Overexpression of PD-L1 was reported to be more common in EBV-positive and MSI-H patients with GC [2,26,34]. In this study, the ORR of MSI-H patients with and without PD-L1 was 66% and 100%, respectively. The ORR of EBV-positive patients with and without PD-L1 was 75% and 0%. Despite the small sample size, patients with MSI-H had better ORR and PFS than patients with MSS, regardless of the level of PD-L1. By contrast, EBV-positive patients with PD-L1 expression had better ORR and PFS compared with patients with PD-L1 CPS < 1. Therefore, PD-L1 expression ≥ 1 may be a useful biomarker for confirming the clinical response of EBV-positive GC. We further validated the relationship of EBV and PD-L1 with the TCGA database. In TCGA, there were 371 gastric cancer patients with RNA-seq gene expression data. Within these patients, EBV was found in 27 patients. Because the TCGA database only collected mRNA expression of PD-L1/CD274 and there was no PD-L1 IHC stain data, we defined PD-L1 high as the top 25% of mRNA expression of PD-L1/CD274 for further analysis, according to a previous study [18]. The EBV-positive patients were divided into two groups: 19 patients with PD-L1 high and eight patients with PD-L1 low. The available, possible predictive factors such as TMB and TILs were analyzed. There was no significant difference of TMB between the two groups (Figure S1). Higher TIL density was found in the EBV-positive and PD-L1 high group, with higher T cells, monocytic lineage, and cytotoxic lymphocytes (Figure S2). This finding could partially explain the better response and longer progression-free survival in patients with EBV-positive and PD-L1 ≥ 1 who received immune check-point inhibitor therapy. However, because of the lack of immunotherapy data in the TCGA database, a further large-scale, prospective clinical trial is still indicated for validation.

This study had several limitations. First, this was a retrospective, single-institute study with a small sample size; recall bias and selection bias may have been present. Second, because immunotherapy is expensive, most patients in our hospital received immunotherapy only when any one of the biomarkers was positive, as well as terminal cases without other effective treatment options. Therefore, few all-negative patients were present in this study, resulting in a relatively small control group. Third, PD-L1 was evaluated using the pharmDx immunohistochemistry assay (PD-L1 IHC 22C3) and based on CPS. This assay is consistent with that of the KEYNOTE trials, but not the CheckMate trials, which used PD-L1 IHC 28–8. This difference may have interfered in the interpretation of PD-L1. However, further evaluation results of the clinical response and survival were consistent with that of the CheckMate trials. Therefore, the interference due to the use of different assays for PD-L1 CPS may be minimal.

## 5. Conclusions

In conclusion, this real-world study demonstrated that PD-L1 CPS ≥ 5, CPS ≥ 10, and MSI-H were independent biomarkers for predicting the efficacy of immunotherapy. In patients with EBER, the better ORR and progression-free survival could be observed only when patients combined with CPS ≥ 1. Furthermore, MSI-H and EBER are mutually exclusive, and the incidence combined with PD-L1 CPS ≥ 1 was high and a better prognosis was observed in these patients. Thus, MSI-H, EBER-positive and PD-L1 expression were useful predictive biomarkers for immunotherapy in gastric cancer.

## Figures and Tables

**Table 1:** Patient characteristics.

Characteristics	*n*	Range/Percentage
Age (years)	65.5	20–93
Sex(male)	23	44.2%
Staging IV	39	75%
ECOG 0–1	48	92.3%
Normal liver function	48	92.3%
Normal renal function	48	92.3%
Biomarkers		
MMR	8	15.4%
EBER	5	9.6%
PD-L1		
≥1	29	55.8%
≥5	9	17.3%
≥10	6	11.5%
Lines of treatment		
1st	10	19.2%
2nd	10	19.2%
3rd or later	32	61.5%
Immunotherapy		
Nivolumab	38	73.1%
Pembrolizumab	12	23.1%
Atezolizumab	2	3.8%
Treatment courses	6	1–64
Previous therapies		
Previous surgery		
Curative	19	36.5%
Palliative	12	23.1%
No surgery	21	40.4%
RT at primary tumor	12	23.1%
Cisplatin	10	19.2%
Oxaliplatin	32	61.5%
5-FU	22	42.3%
Taxanes	27	51.9%

Population: six patients with both MSI-H and CPS ≥ 1 and four patients with both EBER and CPS ≥ 1. No patient had both MSI-H and EBER.

**Table 2:** Outcomes for different biomarkers.

Response	All Negative	CPS1	MSI-H	EBER
CR	0	3	0	1
PR	3	10	6	2
SD	12	3	1	0
PD	5	13	1	2
ORR	15.0%	44.8%	75%	60%
*p*		0.035	0.005	0.07

**Table 3:** Outcomes of patients with different cut-off levels of PD-L1 CPS.

Response	All Negative	CPS ≥ 1	CPS ≥ 5	EBER ≥ 10
CR	0	3	3	3
PR	3	10	3	2
SD	12	3	1	0
PD	5	13	2	1
ORR	15.0%	44.8%	66.7%	83.3%

## Data Availability

The data presented in this study are available on request from the corresponding author. The data are not publicly available due to patient privacy.

## Supplementary Materials

The following supporting information can be downloaded at: https://www.mdpi.com/article/10.3390/cancers14010218/s1, Figure S1. Tumor mutation burden between EBV-positive and PD-L1 high group versus EBV-positive and PD-L1 low group, Figure S2. Tumor infiltrating lymphocytes in EBV-positive and PD-L1 high versus PD-L1 low groups.
