# Supplementary material for: Microsatellite Instability, Epstein–Barr Virus, and Programmed Cell Death Ligand 1 as Predictive Markers for Immunotherapy in Gastric Cancer
Source: Cancers (Basel). 2022 Jan 3;14(1):218. doi: 10.3390/cancers14010218 (PMC8750088; doi:10.3390/cancers14010218)
Supplement: Supplementary file 1 [file cancers-14-00218-s001.zip › cancers-1498418-supplementary.pdf]

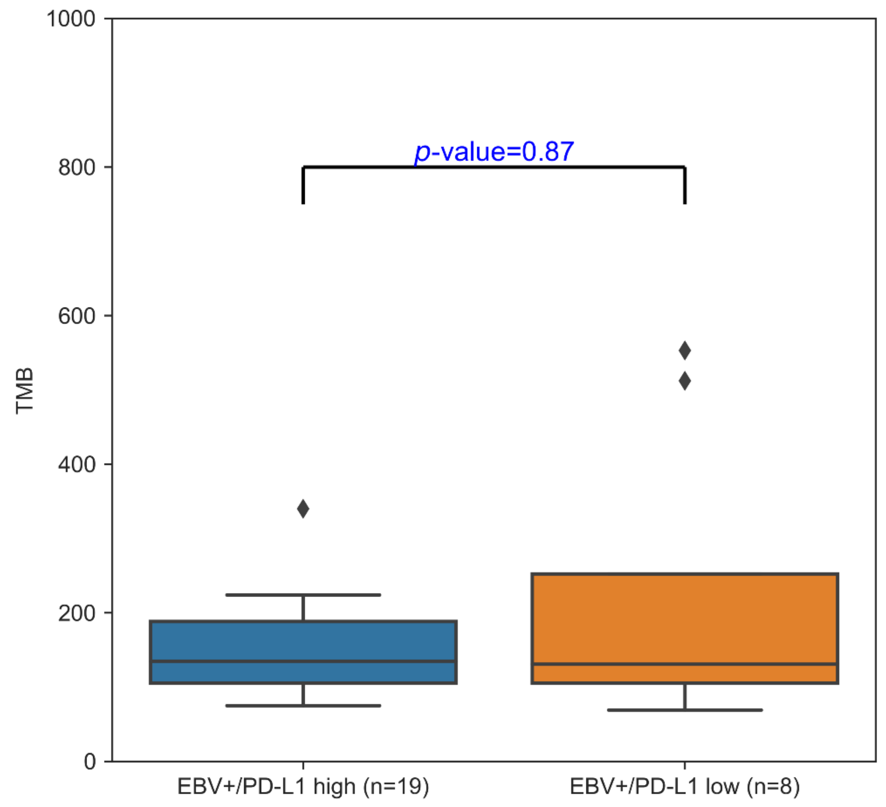


**Figure S1**. Tumor mutation burden between EBV positive and PD-L1 high group versus EBV positive and PD-L1 low group. EBV: Epstein-Barr virus, PD-L1: Programmed death-ligand 1, TMB: tumor mutation burden.

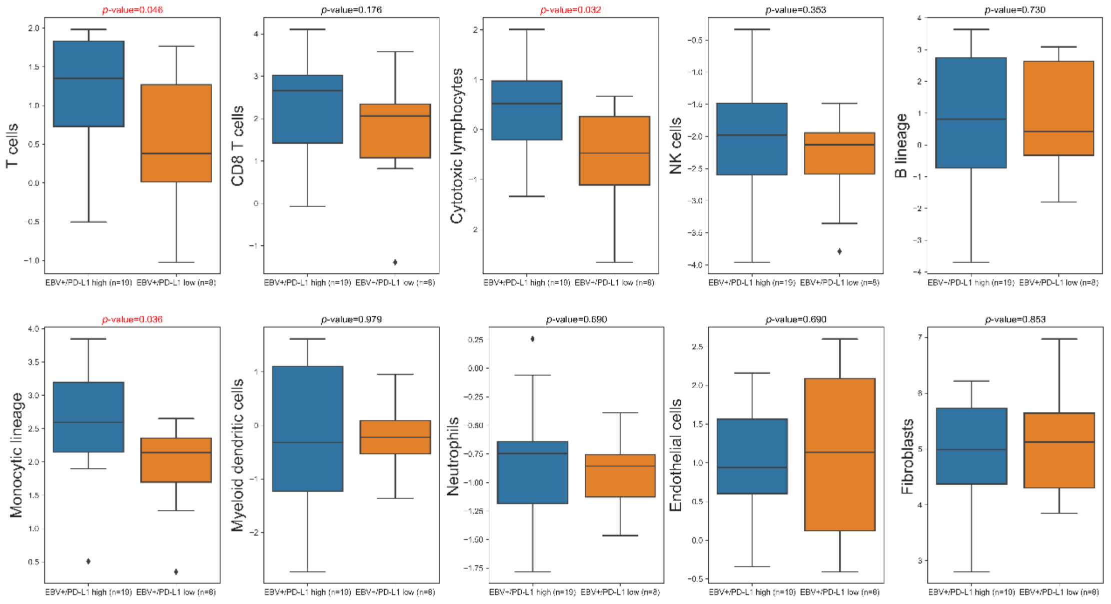


**Figure S2**. Tumor infiltrating lymphocytes in EBV positive and PD-L1 high versus PD-L1 low groups.
